# Tibiofibula Transposition in High-Energy Fractures

**DOI:** 10.1155/2016/6718679

**Published:** 2016-10-11

**Authors:** Peter R. Loughenbury, Rebecca A. Gledhill, Nick Evans

**Affiliations:** Scarborough Hospital, Scarborough, UK

## Abstract

We report two cases of failed attempts at closed reduction of high-energy tibial fractures with an associated fibula fracture. The first case was a 39-year-old male involved in high-speed motorbike collision, while the second was a 14-year-old male who injured his leg following a fall of three metres. Emergency medical services at the scenes of the accidents reported a 90-degree valgus deformity of the injured limb and both limbs were realigned on scene and stabilized. Adequate alignment of the tibia could not be achieved by manipulation under sedation or anaesthesia. Open reduction and exposure of the fracture sites revealed that the distal fibula fragment was “transposed” and entrapped in the medulla of the proximal tibial fragment. Reduction required simulation of the mechanism of injury in order to disengage the fragments and allow reduction. Tibiofibula transposition is a rare complication of high-energy lower limb fractures which has not previously been reported and may prevent adequate closed reduction. Impaction of the distal fibula within the tibial medulla occurs as the limb is realigned by paramedic staff before transfer to hospital. We recommend that when this complication is identified the patient is transferred to the operating room for open reduction and stabilization of the fracture.

## 1. Introduction

High-energy tibial shaft fractures are challenging injuries to treat. They are often open and comminuted and may be associated with other life-threatening injuries [1]. Initial management of these fractures involves realignment of the injured limb and assessment of the neurovascular status. Definitive treatment is then planned based on the fracture type and associated injuries. We report two cases of high-energy tibial fractures that were irreducible despite attempts at closed reduction and required open reduction to restore normal alignment. In both cases the distal fibula was entrapped (“transposed”) within the medulla of the proximal tibial fragment, preventing closed reduction. Interposition of periosteum or soft tissues at the fracture site has been cited as a reason for failed closed reduction on a number of occasions [2–5]. Transposition of the distal fibula into the tibial medulla has not been previously reported in these injuries but can prevent closed reduction and may indicate the need to proceed to immediate operative fixation.

## 2. Case Reports

### 2.1. Case  1

**Figure 1:**
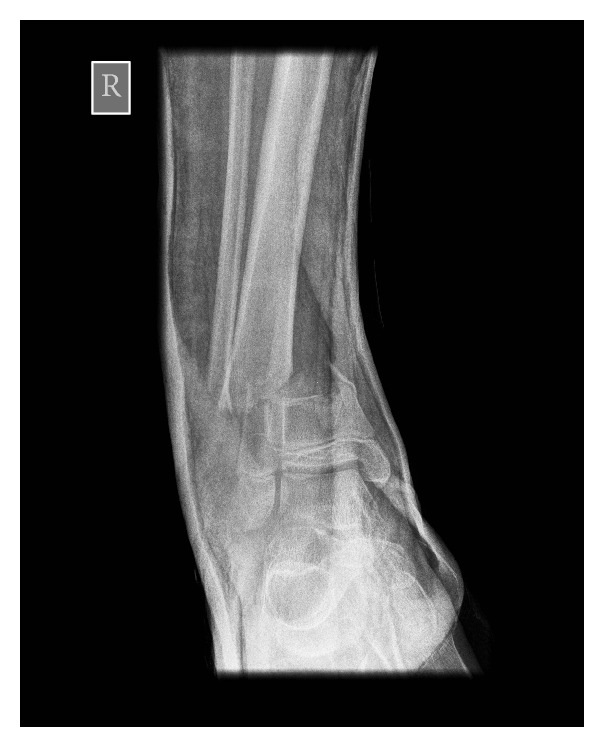
Case one: anteroposterior view of the ankle on admission to the emergency room indicating a tibiofibula transposition.

**Figure 2:**
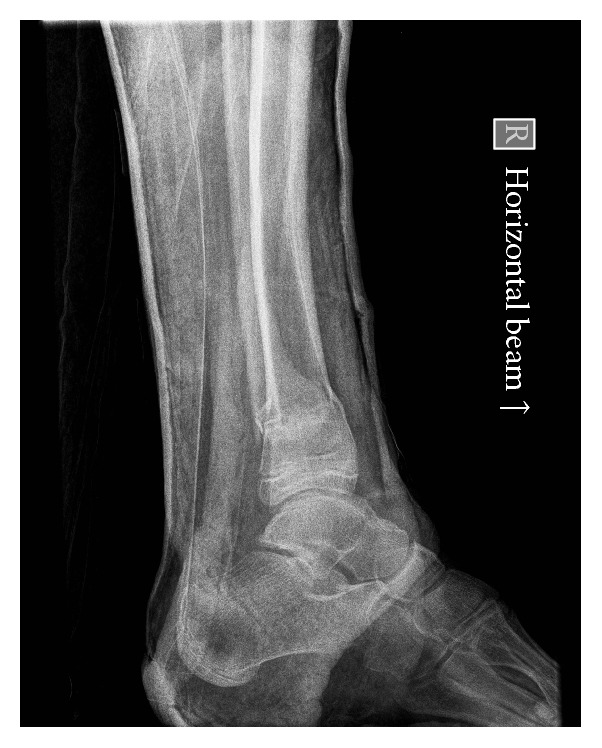
Case one: lateral view of the ankle on admission to the emergency room confirming a tibiofibula transposition.

A 14-year-old healthy male was admitted having fallen from a height of three metres in a derelict building. Paramedics reported a 90-degree deformity of his right leg when they arrived at the scene of injury. The limb was realigned and placed into a “box-splint” for transfer. There was no evidence of neurovascular injury. Plain radiographs revealed a distal tibial metaphyseal fracture with an associated fibula fracture (Figures 1 and 2) and this was the only injury identified during assessment in the emergency room. The injury was closed and reduction was attempted using inhaled nitrous oxide and intravenous opiates. It was not possible to achieve adequate closed reduction either in the emergency room or under a general anaesthetic, with radiological guidance, in the operating room. During open reduction it became clear that the distal fibula fragment was impacted within the medulla of the tibia. In order to reduce the fracture it was necessary to recreate the 90-degree valgus deformity to disengage the fibula from the tibia. Internal fixation of the fracture was performed immediately after open reduction. The patient made a good recovery and has returned to his preinjury levels of activity.

### 2.2. Case  2

**Figure 3:**
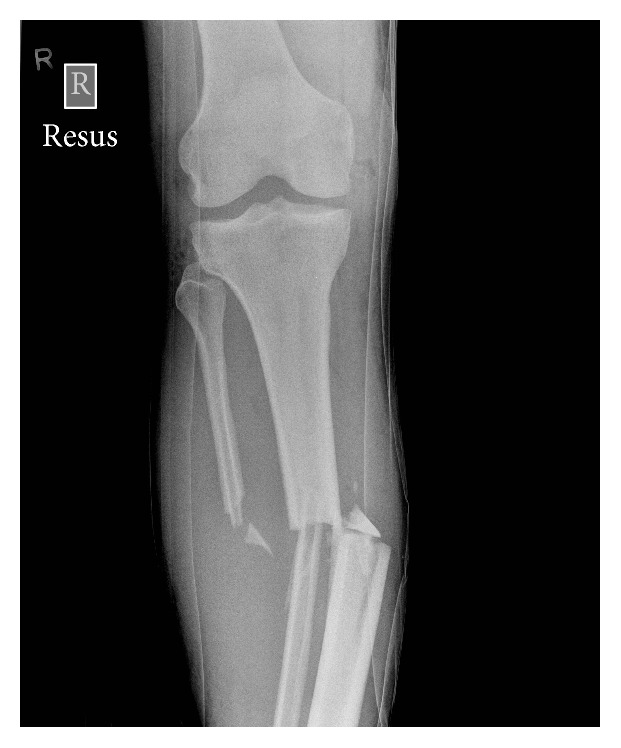
Case two: anteroposterior view of the tibial shaft suggesting a tibiofibula transposition.

A 39-year-old healthy male was admitted with multiple injuries following a high-speed cross-country motorcycle (“Motocross”) accident. On-scene emergency medical services reported a severe 90-degree deformity of the injured limb. The limb was realigned and placed into “box-splint” for transfer to the emergency room. Assessment in the resuscitation room indicated a mid-shaft Gustilo-Anderson [6] Grade IIIA open fracture of the tibia (Figure 3). Other injuries sustained included a closed ipsilateral femoral fracture and the patient was taken to the operating room for immediate wound debridement, fracture reduction, and fixation. Accurate closed reduction of the tibial fracture was not possible. Subsequent debridement of the wound and exploration of the tibial fracture site revealed that the distal fibula fragment was “transposed” into the medulla of the proximal tibia. Replication of the original mechanism of injury was required to release the fibula fragment and allow definitive fixation. The tibial fracture was then stabilized using an intramedullary nailing technique. The postoperative recovery was uneventful and the tibial fracture was united when the patient was reviewed at three months following the injury.

## 3. Discussion

Failure of adequate closed reduction in high-energy tibial fractures requires operative exposure of the fracture site to achieve anatomical fracture reduction. Where closed reduction is not possible it is likely that periosteum or soft tissue is entrapped within the fracture and this may need to be removed. Interposition of soft tissue is a rare complication but has been reported in the literature [2–4]. Grace (1983) [2] reported three cases where the anterior tibial tendon was interposed in paediatric Salter-Harris type II distal tibial fractures, leading to a failure of closed reduction and the need for operative exposure of the fracture site. The posterior tibial tendon may also become entrapped in the fracture site and prevent closed reduction [3, 4]. Failure of closed reduction due to interposed periosteum is more common and is more likely in tibial fractures with greater than 20% displacement and where there is an associated fibula fracture [5]. Where the distal tibial epiphysis is involved anatomical reduction is vital to allow timely closure of the physis [7]. In addition, the close proximity of neurovascular structures at this level makes them prone to simultaneous entrapment at the fracture site. It is therefore important to monitor and document the neurovascular status of the limb both immediately after injury and after any attempts at fracture reduction.

The two cases reported here represent an unusual reason for failure of closed reduction. To our knowledge this has not previously been reported in the literature. In both cases there was a high-energy valgus deforming force applied to the leg. The limbs were realigned by paramedics at the scene of the accident and immobilised in a “box-splint.” We believe that transposition of the distal fibula fragment into the proximal tibia occurred at the time of this realignment prior to transfer to hospital. Realignment of the limb as a first-aid measure remains the correct course of action in order to protect the neurovascular supply and allow safe transfer of the patient to the receiving unit [8]. However, in this case the initial closed reduction appears to have impacted the distal fibula fragment into the medulla of the tibia.

The initial radiographs taken in the emergency room suggest that the distal fibula had been transposed into the tibial medulla. In the first case (Figures 1 and 2) two views confirm this transposition has occurred. However, in the second case (Figure 3) the single view taken prior to transfer to theatre makes it difficult to comment on whether there has been a tibiofibula transposition. This highlights the need for two initial views when dealing with these high-energy injuries. In both cases open reduction of the fracture was required and replication of the 90-degree deforming force was needed to release the distal fibula fragment. We would recommend that where X-rays show that a tibiofibula transposition has occurred, open reduction of the fracture in the operating theatre is required to disengage the distal fibula from the proximal tibia.

## 4. Conclusion

Tibiofibula transposition is a rare complication of high-energy lower limb fractures and may prevent adequate closed reduction. Impaction of the distal fibula within the tibial medulla occurs as the limb is realigned by paramedic staff before transfer to hospital. Two X-ray views of the fracture are required to identify that a transposition has occurred and we recommend that when this is identified the patient is transferred to the operating room for open reduction and stabilization of the fracture.
